# Book Notices

**Published:** 1885-12

**Authors:** 


Otis Clapp & Son's Visiting List and Prescription Record.

This list resembles, somewhat, the lists usually made for physicians of the "regular" school. It differs from most lists by being perpetual. It may be used in any year and at any time of the year without wasting space. It contains the usual obstetrical calendar and a list of poisons and their antidotes. The paper is of excellent quality and the binding neat and substantial.

A New Journal.—With this (November) number the publication of Boericke & Tafel's *Bulletin* will cease, and in its place will be issued *The Homœopathic Recorder* six times a year. Dr. J. T. O'Connor has accepted the editorship. The journal will be devoted mainly to giving excerpts of interesting articles from the foreign medical press, and to the introduction of new remedies and the dissemination of a better knowledge of many of the older ones.

The Homœopathic Physician's Visiting List and Repertory. By Robert Faulkner, M. D. Second Edition. Boericke & Tafel; price, $2.00.

This useful adjunct to the physician's daily practice comes to us in neat binding and gilt edges. It is conveniently arranged and is everything the homœopathist can desire.

